# Quaternized Chitosan Crosslinked Networks for pH-Responsive Macromolecule Delivery: A Review

**DOI:** 10.3390/polym18050649

**Published:** 2026-03-06

**Authors:** Tongtong Wang, Hui Sun

**Affiliations:** 1 School of Light Industry Science and Engineering, Beijing Technology and Business University, Beijing 100048, China; 2330401039@st.btbu.edu.cn; 2 Beijing Key Laboratory of Quality Evaluation Technology for Hygiene and Safety of Plastics, Beijing Technology and Business University, Beijing 100048, China

**Keywords:** quaternary ammonium salt chitosan, green crosslinking agents, crosslinked networks, controlled-release delivery, pH-responsive smart packaging, antibacterial-anticancer synergistic therapy

## Abstract

Chitosan, a biocompatible and biodegradable polysaccharide, exhibits notable antibacterial properties. However, its practical applications are often constrained by inherent limitations such as poor solubility (restricted to acidic media) and suboptimal mechanical strength. By constructing dynamic covalent networks with QCS and green crosslinkers (e.g., genipin, dialdehyde cellulose), materials acquire excellent pH-responsive intelligence. This review elaborates on the molecular design, crosslinking strategies, and applications in intelligent packaging and targeted therapy. The synergistic Schiff-base/hydrogen-bonding mechanism enables dual (pH/enzyme) responsive release. We clarify the relationship between quaternization degree and cytotoxicity as a key challenge for clinical translation and analyze how green crosslinkers are molecular bridges to tailor network properties. The ‘perception-response’ integrated design principle of QCS demonstrates significant potential for intelligent packaging and antibacterial−anticancer synergistic therapy, while addressing key biosafety considerations.

## 1. Introduction

**Figure 1:**
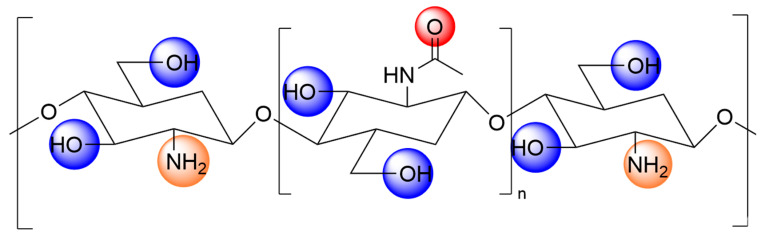
Molecular structure of chitosan.

Chitosan, a natural, high-molecular-weight, basic polysaccharide (Figure 1), is abundantly found in crustacean shells and fungal cell walls. It exhibits favorable properties including antibacterial and antioxidant activity, biocompatibility, and biodegradability [1]. However, native chitosan suffers from limited solubility (being only soluble in acidic solutions), inadequate mechanical properties, and restricted functionality, which curtail its application in targeted drug delivery and food packaging [2]. Notably, recent research findings by Bilican et al. indicate that dissolving chitosan in acetic acid using this traditional processing method may alter its inherent polymer structure, thereby resulting in the loss of its intrinsic beneficial properties, such as hydrophobicity, crystallinity, and fibrous structure [3]. To address these limitations, researchers have developed various chemical and physical modification strategies. Among these, quaternization has emerged as a key focus due to its ability to significantly enhance solubility, antimicrobial properties, and pH-responsiveness.

Quaternary ammonium chitosan (QCS) is a derivative class obtained by introducing quaternary ammonium cationic groups into the chitosan molecular chain. QCS not only retains the inherent advantages of chitosan but also demonstrates substantially improved solubility across a wider pH range and enhanced drug-carrying capacity, providing an ideal platform for constructing smart responsive systems. The superior properties of quaternized chitosan (QCS), as extensively reviewed in [4], including its pH-independent solubility and enhanced charge density, provide an ideal foundation for such intelligent systems. The chemical rationale for quaternization, a key modification strategy to overcome chitosan’s limitations, is well-documented [5]. This study also introduces different crosslinking strategies for chitosan, explains the concept of crosslinking, and highlights the biosafety issues related to the synthesis of crosslinking agents, thereby promoting attention to fully green alternative solutions. While the design of dynamic covalent networks holds great promise, a critical challenge lies in ensuring their structural stability under harsh physiological conditions, such as the varying pH and enzymatic environment of the gastrointestinal (GI) tract.

Notably, Biswas et al. [6] have comprehensively reviewed various chemical modifications of chitosan (e.g., quaternization, carboxylation, and thiolation) and their biological, pharmaceutical, and medical applications, offering a broad perspective on the potential of chitosan derivatives. However, these reviews, including the recent work by Edo et al. [5], often emphasize the breadth of modification techniques rather than providing an in-depth analysis of QCS-based pH-responsive crosslinked networks. Specifically, the intricate interplay between molecular structure (e.g., crosslinking density, dynamic bonds), response mechanisms (e.g., Schiff-base cleavage, hydrogen-bonding modulation), and targeted applications (e.g., intelligent packaging, anti-tumor delivery) remains underexplored. Our work addresses this critical gap by centering on QCS-green crosslinker dynamic networks, systematically clarifying how synergistic Schiff-base/hydrogen-bonding enables dual (pH/enzyme) responsive release, and elucidating how green crosslinkers (e.g., genipin) act as molecular bridges to tailor network properties for enhanced biosafety and functionality.

Among crosslinking strategies, those enabling advanced functionalities like injectability and self-healing represent the forefront. Research on QCS-based hydrogels crosslinked via dynamic covalent bonds has demonstrated excellent injectability and autonomous self-healing properties, which are crucial for minimally invasive applications and enhancing material longevity [7]. Furthermore, alternative processing techniques like electrospinning [8] can be integrated with these crosslinking strategies to create fibrous scaffolds with a high surface area for enhanced cell interaction and drug-loading. For instance, Hu et al. developed stable nanoparticles from stearic acid-grafted chitosan and sodium caseinate, employing glutaraldehyde (GA) and oxidized dextran as crosslinkers, in a sequential protocol (GA first, oxidized dextran second), which effectively protected and sustained the release of curcumin in simulated GI fluids [9]. This work validates the concept but also underscores the inherent biosafety concerns associated with crosslinkers like GA, thereby motivating our investigation into fully green alternatives such as genipin to construct safer intelligent delivery systems.

When active molecules are encapsulated within such a Schiff-base−hydrogen bond crosslinked chitosan network, the Schiff-base bonds can cleave under specific conditions (e.g., acidic environments, enzymatic action), enabling stimulus-responsive release. Moreover, many active molecules contain groups capable of forming hydrogen bonds (-OH, -NH_2_, and -COOH) that interact with hydrogen-bonding sites within the carrier network. This interaction not only stabilizes the encapsulated molecules but also fine-tunes their release behavior, addressing issues such as low water solubility [10], chemical instability, susceptibility to photodegradation/thermal degradation, and rapid metabolic clearance in physiological environments [11].

**Figure 2:**
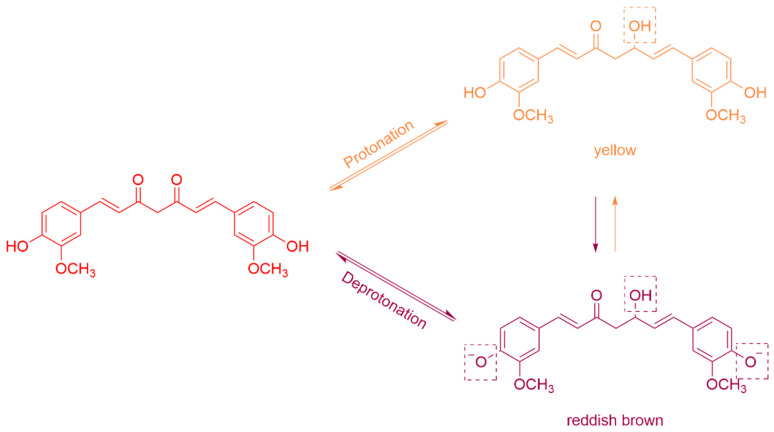
Molecular structure of curcumin and its pH-dependent tautomerization.

Curcumin (CUR), a representative natural polyphenolic compound, possesses unique phenolic hydroxyl and β-diketone structures that confer antioxidant, antibacterial, and pH-responsive color-changing properties. Specifically, its color transitions from yellow to red as the environment shifts from acidic to alkaline (Figure 2) [12]. Consequently, its inherent indicator properties hold considerable promise for applications in chitosan-based smart packaging. This paper systematically reviews the chemical modification strategies of chitosan, the design of crosslinked networks, and their applications in drug delivery, pH-responsive smart packaging, and personal care products. It further discusses the advantages of green crosslinking agents and outlines future challenges and development directions.

Schematic illustration of the structural changes in curcumin during its color-changing reaction in response to pH, transitioning from yellow in acidic conditions to reddish brown in alkaline conditions.

## 2. Chitosan and Its Derivatives

The superior properties of quaternized chitosan (QCS) are rooted in specific chemical modifications. This section first details the primary modification strategies for chitosan, with a focus on the quaternization methods that produce QCS, and their profound impact on material properties.

### 2.1. Chitosan Modification Research

The application of chitosan in controlled release is limited by its uncontrollable swelling in water [13]. This has prompted the development of chemical modifications, with quaternization emerging as a major strategy to endow it with excellent, pH-independent water solubility and key functional properties. Therefore, modifying the amino and hydroxyl groups of chitosan is essential to enhance its properties. Primary modification methods include quaternization, carboxymethylation, grafting, and crosslinking [14] (Table 1).

### 2.2. Preparation and Characteristics of Quaternary Ammonium Salt Chitosan

Among various chitosan modification methods, quaternization has proven to be one of the most effective strategies for constructing intelligent delivery systems, owing to its ability to significantly improve solubility and positive charge. This section elaborates on the specific preparation methods of QCS and its structure−activity relationship.

#### 2.2.1. Chemical Synthesis Methods for Quaternary Ammonium Salt Chitosan (QCS)

**Figure 3:**
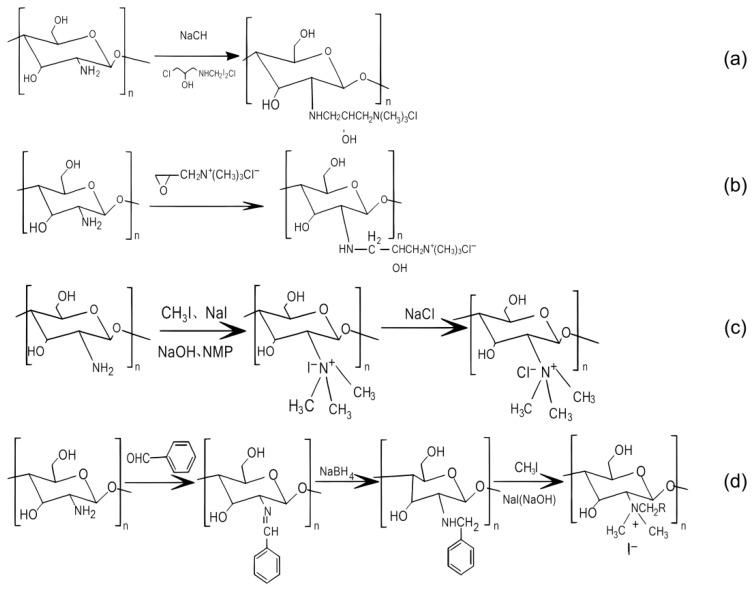
Reaction mechanisms for the synthesis of quaternary ammonium chitosan (QCS). Schematic diagrams of the reaction pathways for: (**a**) direct quaternization using GTA or CHPTAC; (**b**) epoxypropyl quaternization using EPTAC; (**c**) alkylation quaternization using methyl iodide; and (**d**) reductive amination via a Schiff-base intermediate. Key: R represents the chitosan polymer backbone.

Water-soluble quaternary ammonium salt chitosan (QCS) has garnered extensive attention due to its exceptional water solubility and antimicrobial activity [27]. The preparation methods for QCS are diverse, with common approaches including direct quaternization, epoxypropyl quaternization, and alkyl quaternization (summarized in Table 2), as well as typical synthetic reaction mechanisms (Figure 3). As schematically illustrated in Figure 3, the four primary QCS synthesis routes (e.g., direct quaternization using GTA/CHPTAC) proceed via distinct pathways, highlighting the versatility in introducing the quaternary ammonium group onto the chitosan backbone. Among these, the direct quaternization method using reagents such as glycidyl trimethylammonium chloride (GTA) and 3-chloro-2-hydroxypropyltrimethylammonium chloride (CHPTAC) is mostly employed due to its high reaction efficiency and operational simplicity.

#### 2.2.2. Key Factors Influencing the Properties of Quaternized Chitosan

The degree of quaternization (DQ) has multifaceted effects on polymer properties. A higher DQ typically enhances charge density and water solubility but may simultaneously compromise antimicrobial activity and introduce cytotoxicity. While a higher density of quaternary ammonium groups generally bolsters antimicrobial performance, this must be balanced against potential cytotoxic risks. Furthermore, quaternized polymers can exhibit pH-responsive behaviors, such as swelling or shrinkage under specific pH conditions.

##### Influence of Quaternization Degree on Zeta Potential, Water Solubility, and Charge Density

Degree of deacetylation (DQ) exhibits a direct quantitative correlation with zeta potential, water solubility, and antibacterial activity. Studies consistently demonstrate that increasing DQ progressively elevates the zeta potential of chitosan derivatives while correspondingly enhancing water solubility within neutral-to-alkaline pH ranges [34]. A study on phosphonium-functionalized chitosan derivatives with three distinct degrees of substitution (15.6%, 19.8%, and 24.2%) indicates that the zeta potential exhibits a direct positive correlation with the degree of substitution [35]. Similarly, quaternized chitosan-g-cyclodextrin derivative (QCD-g-CS), synthesized by Sajomsang et al., exhibits excellent water solubility, with measured zeta potentials ranging from +25 mV to +40 mV [36]. This study provides experimental evidence that quaternization modification simultaneously enhances both the surface charge and solubility of the material.

Introducing quaternary ammonium groups into the chitosan molecule significantly enhances its water solubility. These permanently charged groups enable the formation of intermolecular hydrogen bonds with water molecules. Research indicates that the water solubility of QCS generally increases with rising DS. For instance, a study modifying chitosan (CS) membranes with 2,3-epoxypropyltrimethylammonium chloride (GTA) identified optimal preparation conditions that significantly enhanced both positive charge density and water solubility [28].

The DS also directly determines the charge density of QCS. A higher DS introduces more quaternary ammonium groups, resulting in a greater positive charge per QCS molecule. Charge density directly governs the interaction strength between quaternized chitosan (QCS) and negatively charged surfaces or molecules. For example, studies have explicitly shown that DS significantly affects the adsorption behavior of QCS on charged surfaces (such as silica) [37].

This increased positive charge density enhances electrostatic interactions with negatively charged bacterial membranes, thereby improving antimicrobial efficacy. Further discussion is provided in the subsequent section.

##### Influence of Quaternary Ammonium Groups on Antibacterial Activity

The antibacterial activity of QCS is heightened by an increased substitution degree (DS) of alkyl trimethylammonium groups on the polymer chain. This translates to exceptional efficacy in practical applications. For example, QCS/tannic acid hydrogels have demonstrated an antibacterial rate of >99% against S. aureusand and significantly promoted wound healing in diabetic rat models [38], providing a direct performance benchmark for antibacterial wound dressings. Beyond antimicrobial applications, QCS-based materials also show promise in rapid hemostasis [39], highlighting the versatility of QCS.

**Figure 4:**
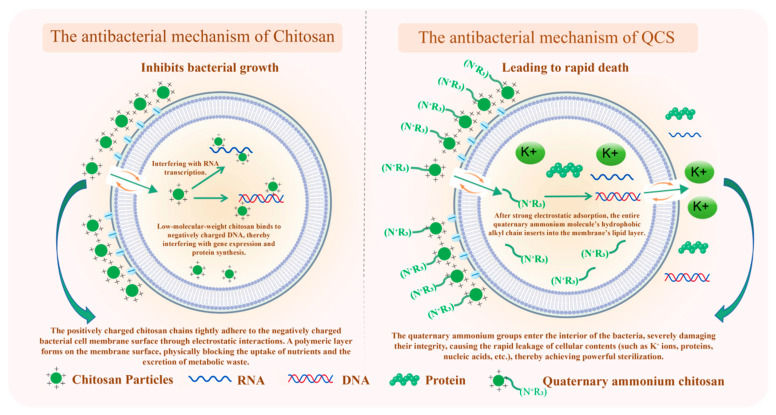
The antibacterial mechanism diagram of chitosan and quaternized chitosan.

The antibacterial effect is mainly achieved through these substituents, which enhance the electrostatic interactions between chitosan and negatively charged components on the bacterial cell wall (such as lipopolysaccharides and teichoic acids), thereby increasing the antifungal activity of chitosan. This electrostatic interaction disrupts membrane integrity, leading to cellular leakage and death [40]. The specific antibacterial mechanism of chitosan quaternary ammonium salts and the differences in the antibacterial mechanism of chitosan are presented in Figure 4.

While antimicrobial activity generally increases with a higher DS [38], an excessively high DS may lead to increased cytotoxicity [41] and diminished mechanical strength [42]. Controlling the DS allows for the modulation of antibacterial activity and biocompatibility. For instance, research by Li et al. indicates that O-quaternized chitosan derivatives with higher DQ values exhibit stronger antibacterial activity against *Staphylococcus aureus* and *Escherichia coli* compared to native chitosan, with the intensity of activity showing a direct positive correlation with zeta potential values. However, there exists a critical threshold beyond which the benefits of increasing the degree of quaternization (DQ) are counteracted by cytotoxicity. Studies indicate that significant cytotoxicity occurs when DQ exceeds 50%, characterized by reduced cell viability and increased lactate dehydrogenase release [43]. Peng et al. further demonstrated this trend, showing that hydroxypropyltrimethyl ammonium chloride chitosan (HACC) with DS 44% exhibited significant cytotoxicity, whereas DS 18% maintained excellent biocompatibility [44]. Therefore, in biomedical applications, to balance enhanced functionality with acceptable biocompatibility, the optimal range of DQ is typically recommended to be set between 20% and 40%.

Beyond acute cytotoxicity, the in vivo degradation and long-term fate of QCS networks are critical for clinical translation. The primary degradation pathway involves lysozyme-mediated hydrolysis of the β-1,4-glycosidic bonds in the chitosan backbone, generating oligosaccharides and glucosamine residues that enter metabolic pathways [4,7]. Higher DQ slows this process due to increased steric hindrance [7]. However, the metabolic fate of the quaternary ammonium moiety itself remains unclear and may exist as small molecular fragments and accumulate in tissues. Liu et al. reported that an amphiphilic QCS derivative (DS = 15.58%) exhibited a plasma half-life of 48 h and accumulated predominantly in adipose tissue and the gastrointestinal tract [45]. Hemocompatibility also correlates with DQ: While moderate DQ (15–20%) shows acceptable safety, DQ exceeding 40–50% induces significant hemolysis due to excessive positive charge density, disrupting red blood cell membranes [46,47]. These findings reinforce the importance of DQ optimization (20–40%) not only for balancing antibacterial efficacy with acute cytotoxicity, but also for ensuring favorable long-term biocompatibility and hemocompatibility.

Furthermore, cationic charge density is a critical factor for drug delivery carriers, directly influencing electrostatic interactions with macromolecules and determining loading efficiency [48]. Materials exhibiting higher quaternary ammonium charge density typically demonstrate superior antibacterial efficacy.

QCS plays an important role in applications such as antimicrobial coatings [49], biomaterials [27], water treatment flocculants [50], and as vaccine adjuvants. The antibacterial and anti-biofilm properties are closely related to its molecular structure; QCS derivatives with different spacer groups exhibit distinct activities [51], with specific discussions presented in Section 4.

Notably, this DS-dependent balance between antibacterial activity and toxicity provides a crucial structure−activity relationship theoretical foundation for the application design discussed in subsequent chapters. Reducing cytotoxicity while maintaining antibacterial activity has emerged as the key breakthrough for advancing QCS materials from basic research to clinical applications.

##### pH-Responsiveness (Swelling/Shrinkage Behavior)

Chitosan is a polysaccharide containing abundant amino (-NH_2_) and hydroxyl (-OH) groups along its molecular chain [1]. Under acidic conditions, these amino groups undergo protonation to form -NH_3_^+^, imparting a positive charge to chitosan and thereby enhancing its water solubility and swelling properties. As the pH increases, the amino groups deprotonate, reducing chitosan’s solubility and swelling capacity. This process induces matrix shrinking through mechanisms such as molecular chain aggregation, dehydration, and network collapse.

The introduction of quaternary ammonium groups further modulates chitosan’s pH-responsiveness. These permanently charged groups enhance chitosan’s water solubility across a broader pH range and strengthen its affinity for negatively charged substances [52]. In QCS-based hydrogels under acidic conditions, the remaining amino groups protonate, generating electrostatic repulsion that promotes expansion of the matrix network. This facilitates greater water absorption and leads to gradual swelling. Under alkaline conditions, the amino groups deprotonate, disrupting ionic interactions and potentially facilitating the formation of intramolecular or intermolecular hydrogen bonds. This hydrogen-bonding restricts matrix network swelling, expels water, and induces contraction [39].

The pH-responsive swelling/shrinkage behavior of QCS is a crucial property. By regulating its chemical structure and environmental factors, precise control over matrix performance can be achieved to meet diverse application requirements. QCS further combines this inherent pH-responsiveness with its crosslinkable characteristics, enabling the construction of structurally stable smart materials sensitive to specific pH environments, which confer unique advantages in biomaterials.

## 3. Tailoring Network Properties Through Crosslinking Strategies

**Figure 5:**
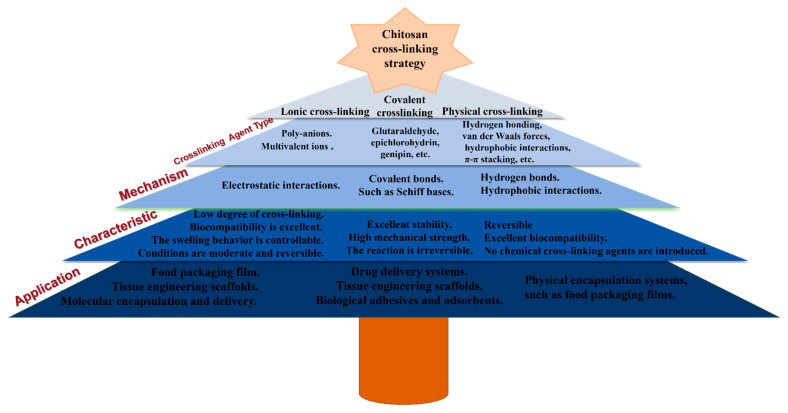
Crosslinking strategies for chitosan: types, mechanisms, and applications.

Building on the chemical structure of QCS, a key strategy to control its functionality is the engineering of network properties through crosslinking. This approach is fundamental because, in fields such as drug delivery and packaging, the topological structure of crosslinked networks (e.g., crosslink density, pore size) directly influences critical performance metrics like drug-loading efficiency and release kinetics. These network properties, in turn, determine a spectrum of crucial functionalities, including mechanical strength, swelling behavior, intrinsic pH-responsiveness, and drug release characteristics, as systematically summarized in Figure 5.

### 3.1. Crosslinking Strategies and Performance Regulation

The excellent performance of QCS lies in its crosslinking and fixation into a stable network structure. The choice of crosslinking strategy directly determines the network topology, which, in turn, governs its controlled-release behavior. Some crosslinking methods can effectively enhance the mechanical strength and stability of chitosan. According to their mechanisms of action, they can be roughly divided into ionic crosslinking, covalent crosslinking, and physical crosslinking, with specific mechanisms of action shown in Figure 5.

Green crosslinking agents (e.g., genipin, diacetyl cellulose) are favored for their excellent biocompatibility and low toxicity. The pursuit of green processing strategies extends beyond crosslinking to the fabrication of nanocarriers. A notable example is the solvent-free, pH-driven method developed by Lei Wang et al. [53], which enables the self-assembly of caseinate/zein nanocomposites for curcumin encapsulation and subsequent edible film formation. This approach aligns with the green crosslinking philosophy by eliminating organic solvents, offering a sustainable pathway for intelligent material preparation.

Quaternary ammonium chitosan (QCS), when blended with such crosslinkers, allows covalent bonds, electrostatic interactions, and hydrogen bonds to synergistically form a three-dimensional dynamic crosslinked network. The mechanical strength, stability, and biocompatibility of materials can be significantly improved by such network structures. This dynamic structure exhibits pH-responsiveness: For instance, under acidic conditions, covalent Schiff-base bonds readily hydrolyze, leading to network disintegration. Such intelligent networks hold broad application prospects in drug delivery and tissue engineering. For example, hydrogels prepared by crosslinking cellulose with chitosan demonstrate excellent biocompatibility and degradability, promoting wound healing [54]. As another method to improve the properties of chitosan films through crosslinking via imine bonds, this study is the first to synergistically utilize the hydroxyl/amide groups of PHEAA and the amino groups of CS for Fe^3+^ chelation. Compared with pure CS adsorbents (typically <20 mg/g), the adsorption capacity of this material is increased by more than 80%, and the mechanical strength is improved by three times. A thin film with both high-efficiency iron adsorption and blood safety has been developed [55].

Other natural crosslinking agents, such as genipin, exhibit favorable biocompatibility and low toxicity [56]. Genipin crosslinks with the amino groups of chitosan to form stable networks, enhancing the material’s mechanical properties and biocompatibility [57]. Citric acid, as a natural organic acid, undergoes esterification reactions with the amino and hydroxyl groups of chitosan to form crosslinked structures. Citric acid-crosslinked chitosan materials exhibit excellent biocompatibility and degradability, making them suitable for applications such as food packaging and sustained release systems [58]. Other studies have employed tannic acid for physical/chemical dual crosslinking. Curcumin was clearly loaded, and glucose-sensitive controlled release was achieved [59].

To put the present survey into perspective, Mottaghitalab et al. recently compiled an overview focused exclusively on genipin- and dialdehyde-cellulose-crosslinked quaternized chitosan hydrogels for wound healing [7]; here, we extend this green crosslinking paradigm to oral anticancer delivery and intelligent food packaging, and further quantify structure–performance relationships using Flory–Rehner analysis. Therefore, the selection of crosslinking agents requires a comprehensive consideration of material properties, application requirements, and reaction conditions, alongside the precise regulation of crosslinking performance. Among these factors, crosslink density is a key parameter influencing the performance of the crosslinked network. An appropriate crosslinking density enhances the material’s impact strength and toughness [60], allowing for the customization of properties such as hardness, elastic modulus, and swelling behavior. In biomedicine, crosslinking density significantly influences the mechanical properties, degradation rate, and drug release behavior of hydrogels [61].

Swelling experiments serve as a crucial method for characterizing network structures of crosslinked polymers, particularly for determining crosslink density [62]. Swelling behavior is closely linked to structural properties of the polymer network, including crosslink density, network topology, and polymer−solvent interactions [63].

Beyond conventional crosslinking density regulation, emerging studies have emphasized that the mesoscale ordering within chitosan networks can further modulate responsive behavior. For instance, controlled reconstruction of chitosan network architecture via interfacial processing strategies has been reported to regulate polymer chain ordering and enhance pH-responsive structural stability, highlighting the importance of network organization beyond simple crosslink density parameters. Such findings provide additional insight into structure−property relationships in chitosan-based systems and offer expanded design possibilities for QCS materials [64].

The Flory−Rehner theory provides a classical framework for estimating crosslink density from swelling data [62]. This theory, grounded in the statistical mechanics of polymer chains, relates the degree of swelling to the crosslinking density of the network. At swelling equilibrium, the elastic recoil force of the polymer network balances the osmotic pressure of the solvent. The Flory−Rehner equation is expressed as:

(1) −ln1−V2−V2−XV22=V1nV213−V22

where *V*_2_ denotes the volume fraction of the polymer at swelling equilibrium, *X* represents the polymer−solvent interaction parameter, *V*_1_ is the molar volume of the solvent, and *n* is the effective number of crosslinks per unit volume (correlating with crosslink density).

By determining *V*_2_ through swelling experiments and using known values of *V*_1_ and *X,* the crosslink density *n* can be calculated. This parameter is directly related to mesh size and the molecular weight between crosslinking points, and can be used to predict whether drugs of specific molecular weights can be loaded and released from the network [56].

### 3.2. Synergistic Mechanisms of Crosslinked Networks and Their Applications in the Pharmaceutical Field

QCS-based crosslinked networks exhibit remarkable synergistic effects in drug delivery due to their multi-level architecture and intelligent responsiveness to biological environments. This synergy manifests not only in the efficient loading and protection of active molecules but, crucially, in enabling precise, controlled drug release at specific pathological sites, significantly enhancing therapeutic efficacy while reducing side effects.

#### 3.2.1. Synergistically Enhancing Loading, Protection, and Delivery Efficiency

The crosslinked network primarily based on QCS achieves controlled release through structural regulation. The stable three-dimensional network formed between QCS and crosslinkers creates an adjustable barrier, thereby regulating drug diffusion. A higher crosslinking density typically reduces the network pore size, resulting in a slower release rate [65]. This principle is exemplified by nanoparticles prepared through ionic crosslinking of chitosan with sodium tripolyphosphate (TPP) or phytic acid, wherein increased crosslinker concentration resulted in enhanced curcumin encapsulation efficiency and prolonged release profiles [66]. Similarly, chitosan−gelatin composite carriers demonstrate crosslinking-dependent modulation of drug solubility, release rate, and mechanical strength through crosslinking, while also exhibiting excellent antioxidant and antibacterial properties [67].

**Figure 6:**
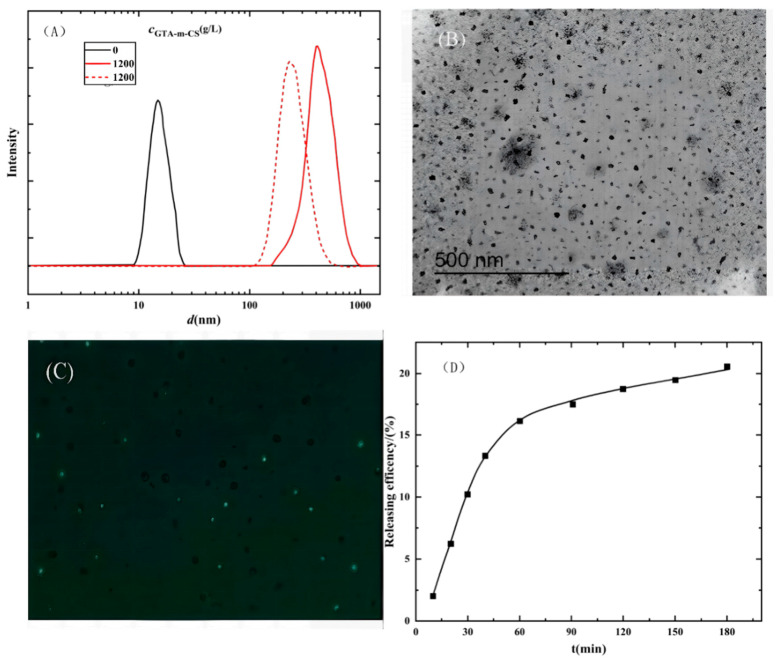
Characterization and curcumin release profile of crosslinked micelles. (**A**) Hydrodynamic radius distribution of micelles. (**B**) Transmission electron microscopy (TEM) image revealing the spherical morphology of the micelles. (**C**) Fluorescence confocal microscopy image confirming the successful encapsulation of curcumin. (**D**) In vitro release profile of curcumin from DDEAC and GTA-m-CS/DDEAC micelles over time, demonstrating sustained release behavior. Reproduced from Ref. [70] under the terms of the CC BY 4.0 license.

Beyond diffusion control, the multi-level architecture of crosslinked networks optimizes drug-loading capacity through multiple synergistic interactions. Functional groups within the network (e.g., amino, carboxyl) efficiently bind macromolecules via electrostatic adsorption or hydrophobic interactions. The high-density quaternary ammonium cations (-N^+^(CH_3_)_3_) on the QCS chain exhibit strong electrostatic adsorption with negatively charged drug molecules (such as the phenolic hydroxyl groups of curcumin), significantly enhancing encapsulation efficiency and drug-loading capacity [68]. This enhancement stems from the increased number of specific active sites, thereby improving the material’s adsorption performance [69]. As illustrated in Figure 6, a system using GTA-modified chitosan and a quaternary ammonium surfactant achieved a curcumin encapsulation efficiency of approximately 32% and sustained release over 180 min, demonstrating potential for pharmaceutical applications [70].

By modulating crosslink density and analyzing the resulting data with these models, the diffusion rate and release profile of the drug can be precisely understood and controlled, enabling sustained release over periods ranging from hours to days and overcoming the burst release issue of traditional formulations.

The crosslinking network leverages its dense structure to provide multiple protective barriers for the drug, effectively shielding it from moisture, oxygen, light, and heat [71], thereby slowing down hydrolysis, oxidation, and photothermal degradation processes. The positive charge of QCS also promotes adsorption of the nanosystem onto negatively charged intestinal mucosal surfaces via electrostatic interactions, prolongs retention time, and enhances intestinal absorption efficiency and oral bioavailability [72].

Our strategy of modulating the release profile by tuning the crosslinking density of green agents finds a compelling parallel and a performance benchmark in the work of Hu et al. [9]. Their study revealed that the sequence of crosslinking, i.e., applying small-molecule GA prior to macromolecular oxidized dextran, was essential to constructing a hierarchically structured network. This specific architecture provided the mechanical robustness to withstand gastric fluid while allowing controlled release in the intestine, achieving a high encapsulation efficiency (>97%) for curcumin. In contrast, our system employs the biocompatible genipin as a single crosslinker, seeking to achieve comparable release kinetics through a simpler strategy. This approach not only leverages a comparable Schiff-base mechanism for pH-responsiveness but also circumvents the potential cytotoxicity associated with GA. The excellent GI stability and release profile demonstrated by their system [9] thus serve as a valuable target for evaluating the efficacy of our green crosslinking approach. As shown in Figure 6, the spherical morphology and uniform size distribution of these crosslinked micelles (Figure 6A,B) ensured efficient cellular uptake (Figure 6C). Most importantly, the in vitro release profile (Figure 6D) confirmed a sustained and prolonged curcumin release over 180 min, a characteristic highly desirable for controlled drug delivery. This demonstrates that our green crosslinking approach provides a simplified and potentially safer strategy for constructing effective delivery systems, achieving comparable functionality while circumventing the need for cytotoxic crosslinkers employed in previous multi-step methodologies.

This encapsulation efficiency can exceed that of conventional micelles and liposomes [73]. The release kinetics of such systems often conform to the Korsmeyer−Peppas or Weibull models [74]. These models are instrumental in quantifying how the modulation of crosslink density, as mentioned above, influences the drug release profile. The fundamental equation of the Korsmeyer−Peppas model is:

(2) $$\frac{M_{t}}{M_{\infty }}=kt^{n}$$

where *M_t_* is the amount of drug released at time *t*, *M*_∞_ is the total drug amount, *k* is the release rate constant, and *n* is the release exponent indicative of the release mechanism (for spherical swellable devices like QCS hydrogels, an *n* value ≤ 0.43 indicates Fickian diffusion, while 0.43 < *n* < 0.85 suggests anomalous transport [75], a coupled process of diffusion and polymer relaxation which is typical for pH-responsive networks). This provides a quantitative means to link crosslinking density (which affects polymer relaxation) to the release mechanism.

The Weibull model is expressed as:

(3) $$\frac{M_{t}}{M_{\infty }}=1-e^{\left(-{\left(\frac{t}{\tau }\right)}^{\beta }\right)}$$

where *β* is the shape parameter and *τ* is the scale parameter. Its flexibility allows it to accommodate diverse release patterns, making it particularly useful for describing the complex release kinetics from QCS networks with heterogeneous crosslinking densities.

The shape parameter *β* of the Weibull model reflects network heterogeneity: *β* = 1 denotes a homogeneous network, *β* < 1 indicates an initial rapid release, and *β* > 1 signifies the presence of a lag period [76]. Combined with the structural parameters derived from the Flory−Rehner theory, these models assist researchers in rationally designing network structures according to target release behavior [74]. To illustrate the practical application of these kinetic models and the diversity of chitosan−based delivery systems, Table 3 summarizes representative examples from the literature, highlighting their encapsulation efficiencies, release conditions, and corresponding kinetic parameters. These data not only demonstrate the tunability of release profiles through crosslinking strategies but also provide quantitative insights into the release mechanisms (e.g., Fickian diffusion vs. anomalous transport) as derived from the Korsmeyer−Peppas exponent *n*. The Weibull shape parameter *β* further reveals the heterogeneity of network structures, guiding the rational design of carriers with desired release characteristics.

Collectively, these examples demonstrate that chitosan quaternary ammonium salts can enhance delivery performance through diverse synergistic mechanisms of crosslinked networks. For instance, diffusion control is achieved by regulating network size; drug-loading capacity is increased via electrostatic and hydrophobic interactions between chitosan and its derivatives with crosslinkers; and drug protection is realized through physical barriers and mucosal adhesion properties. The interplay between the crosslinking density of chitosan quaternary ammonium salts, network structure, and functional group chemistry provides a multifunctional platform for customizing delivery systems to meet specific therapeutic needs.

#### 3.2.2. Construction of pH-Sensitive Crosslinked Network

Based on the principles of dynamic covalent chemistry, QCS-based crosslinked networks can be precisely engineered to respond to specific pH stimuli, thereby enabling intelligent and targeted drug release. The fundamental mechanism primarily relies on Schiff-base bonds that undergo acid-triggered cleavage, as well as hydrogen bond networks that provide structural integrity and environmental shielding.

In quaternized chitosan materials, unreacted amino groups, hydroxyl groups, and ether bonds can form Schiff-base bonds and intramolecular hydrogen bonds with aldehyde groups from crosslinkers. The Schiff-base bonds confer pH-responsiveness, cleaving under acidic conditions. Environments such as tumors (pH~6.5), inflammation, or intracellular endosomes/lysosomes (pH 4.5~6.0) lead to network dissociation or swelling, thereby triggering drug release. Meanwhile the hydrogen bond network provides structural integrity and shields encapsulated active molecules (e.g., drugs, probiotics, essential oils, enzymes, and vitamins) from external degradation [84]. This unique physicochemical structure grants the network exceptional protective and encapsulation capabilities, making it an ideal carrier system for pH-responsive smart release applications. This dual-mechanism design distinguishes QCS networks from single-stimulus responsive systems and enables operation across diverse pH environments, including gastric (pH~2), tumor (pH~6.5), and food spoilage (NH_3_-triggered alkaline) conditions.

The mechanism is enhanced by the network’s dynamic swelling behavior. Under acidic conditions, the protonation of amino groups in chitosan causes the network to swell. Network swelling increases the contact area between the embedded indicator molecules and the environment, leading to more pronounced and rapid color changes. Conversely, under alkaline conditions, amino groups deprotonate, causing the network to contract. Network contraction slows the diffusion of ions and the rate of color change, thereby improving the indicator’s stability [85].

This principle has been successfully applied in pH intelligent indicator packaging. Common pH indicators, such as phenol red, anthocyanins, curcumin, methyl red, and bromothymol blue, function by undergoing a transition between protonated and deprotonated states, resulting in visible color changes across different pH levels [86,87,88,89]. By encapsulating these indicators within a QCS crosslinking network, the material synergistically combines chitosan’s innate pH sensitivity with the regulatory effect of the crosslinked matrix. This integration achieves both “sensing” of external pH changes and an intelligent “response” [90]. For instance, research has incorporated cactus pear extract (CPE) into quaternary ammonium chitosan (QAC)/polyvinyl alcohol (PVA) blends to develop advanced films with antioxidant, antibacterial, and ammonia-sensitive properties. The addition of CPE significantly enhanced these functionalities. As a water-soluble nitrogen-containing pigment, CPE enables the detection of microbial spoilage; the film’s color shifts from purple to orange in response to ammonia produced during food degradation, positioning it as a promising smart-packaging material [91].

Similarly, lithospermic acid (SKN) loaded into quaternized chitosan (HACC)/polycaprolactone (PCL) nanofiber composite films resulted in materials with excellent antibacterial, hydrophobic, barrier, and mechanical properties. These films also exhibited a clear pH-responsive color change from red (pH 2) to blue-violet (pH 12) [92].

Beyond packaging, this technology extends to visual freshness indicators. Research utilizing chitosan-grafted gelatin (CS-g-GEL) hydrogels incorporated with the pH-responsive dye bromothymol blue (BTB) created a colorimetric sensor to monitor chicken breast spoilage. Such studies demonstrate the vast potential of intelligent, visual indicators for real-time food-quality monitoring [93].

The targeting capabilities of these networks can be further refined through molecular design. For example, synthesizing folic acid−chitosan conjugates (FA-CS) and using them to prepare nanoparticles via ionogelation technology (PC-CS/FA-NPs) enhanced the stability and bioavailability of proanthocyanidins during gastrointestinal digestion, while also protecting against oxidative damage [80].

Another strategy to enhance pH-responsiveness involves grafting pH-sensitive polymers onto QCS. Introducing groups like poly(2-(diisopropylamino)ethyl methacrylate) (PDPA), which protonates and becomes positively charged at lower pH, can promote drug release from the carrier in acidic environments like tumors [65]. The sensitivity and functionality of these responsive systems can be further augmented by incorporating nanomaterials such as quantum dots or metal nanoparticles [94].

The construction of pH-responsive crosslinked networks using quaternized chitosan provides a versatile and powerful platform for developing advanced functional materials. Continued exploration of factors influencing these networks will be crucial for optimizing their stability and performance for specific applications.

The design of pH-sensitive networks can be further sophisticated to achieve temporally programmed release, addressing complex therapeutic scenarios that require multiple drugs to act in a specific sequence. A prime example is a core–shell hydrogel system engineered for the sequential delivery of an antiemetic and a chemotherapy drug [95]. In this architecture, the shell layer, constructed with a loose, pH-labile network (e.g., via Schiff-base bonds), rapidly releases an antiemetic in acidic environments to provide immediate symptomatic relief. Meanwhile, the core compartment, composed of a denser or differently crosslinked network, provides sustained and delayed release of the chemotherapeutic agent. This spatially and kinetically differentiated design leverages the foundational pH-responsiveness of dynamic covalent networks to enact a pre-programmed “release logic”, moving beyond single-molecule delivery towards intelligent, combinatorial therapy. Such systems directly illustrate how modulating network architecture (e.g., core vs. shell crosslinking density) can dictate precise release profiles, a theme explored in the following section.

#### 3.2.3. Influence of Crosslinking Density on Drug Delivery Performance

Crosslinking density is a pivotal parameter influencing the performance of drug delivery systems, critically affecting drug-loading capacity, mechanical strength, and degradation rate of the polymeric network [96]. The crosslinked structures formed within polymers not only enhance the mechanical strength and chemical stability of films but also are the primary mechanism enabling controlled release. By precisely adjusting crosslink density and film architecture, sustained release of loaded molecules, such as curcumin, can be achieved [97].

The relationship between crosslinking density and mechanical stability, drug-loading capacity, and brittleness may be explained mechanistically from three perspectives: free volume theory, network topology, and stress distribution. According to the free volume theory, the introduction of crosslinking points consumes the free volume available for polymer chain motion. As the crosslink density increases, the segmental motion capability of polymer chains becomes progressively restricted, thereby enhancing modulus and mechanical strength [60,98]. However, the reduction in free volume simultaneously diminishes the space available for accommodating drug molecules. For macromolecular drugs, the network pore size (ξ) decreases with increasing crosslinking density. When the pore size becomes smaller than the drug’s hydrodynamic radius, macromolecular drugs are physically excluded, thereby reducing the drug-loading capacity [71]. From a network topology perspective, at low-to-moderate crosslinking densities, polymer chains can redistribute stress through segmental rearrangement, thereby conferring toughness and flexibility to the material. As crosslink density increases, the network becomes more uniform and constrained. However, excessively high crosslink density introduces localized stress concentrations at the crosslinking points [99]. These stress concentration points serve as nucleation sites for crack propagation under mechanical loading, explaining the embrittlement observed at high crosslink densities. The critical crosslink density at which the ductile−brittle transition occurs depends on the inherent flexibility of the polymer backbone and the molecular weight (Mc).

The aforementioned inherent trade-offs necessitate optimization according to specific application scenarios. For oral drug delivery systems, an optimal degree of crosslinking enhances the stability of the polymer network, preventing premature drug leakage during preparation and storage. Conversely, an excessively dense network reduces the available free volume and mesh size, constricting the space available for drug molecules to embed and thereby diminishing the overall drug-loading capacity. This limitation is particularly pronounced for larger macromolecular drugs [71].

For tissue engineering scaffolds, load-bearing capacity and dimensional stability are paramount. Consequently, high crosslinking density is required to achieve the target elastic modulus, yet the concomitant reduction in toughness and diminished cell infiltration capacity must be compensated for through structural design. For instance, incorporating macroporous structures via freeze-drying or 3D printing enables the attainment of sufficient mechanical strength while maintaining high porosity. Research by Gonçalves et al. demonstrated that by adjusting chitosan concentration, genipin crosslinker concentration, and freeze-drying temperature (−20 °C), scaffolds with compressive strength up to 0.40 MPa, compressive modulus up to 3.57 MPa, and porosity exceeding 80% could be prepared that are suitable for cancellous bone regeneration [100]. Alternatively, a dual-crosslinking strategy combining covalent crosslinking with physical crosslinking or interpenetrating networks may partially decouple mechanical strength from brittleness. Huang et al. reported a dual-crosslinked pure chitosan hydrogel based on synergistic interactions between a chemical covalent network, hydrophobic interactions, intermolecular hydrogen bonds, and chitosan microcrystalline formation that exhibited approximately two-fold increases in fracture energy, maximum compressive stress, and elastic modulus (7.733 J m^−2^, 10.81 MPa, and 1.33 MPa, respectively), while maintaining low cytotoxicity, demonstrating broad application prospects in tissue engineering [101]. The Flory−Rehner theory (Equation (1)) and rubber elasticity theory provide a quantitative framework for predictive design. By correlating crosslink density with Mc, pore size, and mechanical modulus, researchers can select target parameters according to application requirements, thereby rationally optimizing crosslinking conditions.

**Figure 7:**
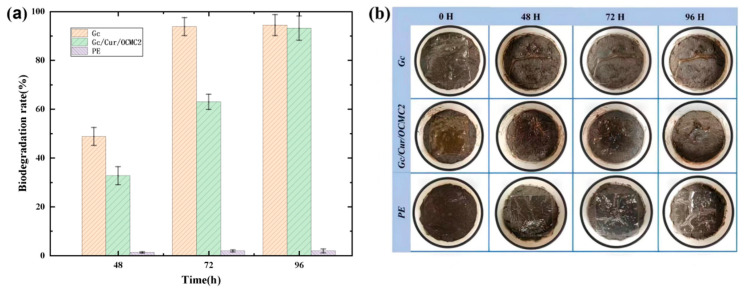
Biodegradation profile and proposed mechanism of the crosslinked gelatin/curcumin film. (**a**) Quantitative biodegradation rate of the film at different time points. (**b**) Schematic illustration of the biodegradation process of the film sample over time. Reprinted with permission from Ref. [102]. Copyright © Elsevier 2025.

The degree of crosslinking can also directly impact the degradation performance of the material. Research utilizing oxymethylcellulose (OCMC) as a green crosslinker to produce curcumin-loaded gelatin films demonstrated that the crosslinked films (Gc/Cur/OCMC2) exhibited excellent mechanical strength, moisture resistance, and antioxidant properties. As illustrated in Figure 7, the crosslinked bonds between chitosan and curcumin enhanced molecular compactness, resulting in a slower degradation rate (63.4% after 72 h) compared to less crosslinked formulations, which ultimately also decomposed into water and carbon dioxide [102].

In addition to modifying the degradation rate of materials, crosslinking density can also alter the fundamental degradation mechanism. Hydrogels with lower crosslinking density may primarily degrade via surface erosion, whereas those with higher crosslinking density are more likely to undergo bulk degradation [103]. The crosslinking points effectively hinder the scission and dissolution of polymer chains, thereby prolonging the material’s lifetime in vivo. This property can be exploited for colon-specific drug delivery, as chitosan and its derivatives are susceptible to degradation by specific enzymes (e.g., glucanases) produced by colonic bacteria, facilitating targeted drug release at the desired site [104]. For instance, a system featuring a curcumin−cyclodextrin inclusion complex as the core, encapsulated by a shell of low-molecular-weight chitosan and unsaturated alginate, successfully achieved enzyme-triggered controlled drug release in the colon [104]. The applications of drug delivery utilizing crosslinked networks will be discussed in detail in the subsequent chapter.

## 4. Application Frontiers and Translational Hurdles

**Figure 8:**
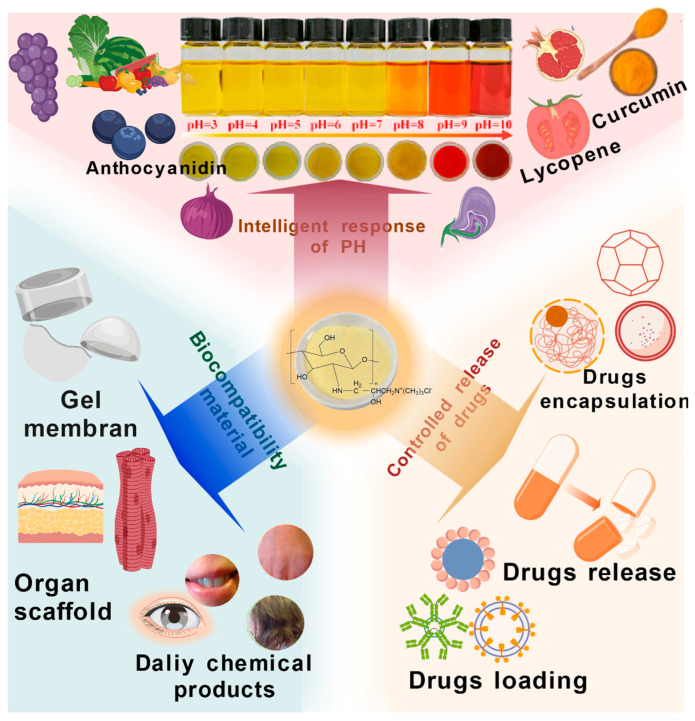
The common application scope of quaternary ammonium salt chitosan.

The intelligent and tunable nature of QCS crosslinked networks, as detailed in the previous chapters, opens the door to a range of advanced applications. As reviewed in [5,105], QCS-based systems have demonstrated significant potential in both biomedical and packaging fields. However, the path to clinical and commercial translation requires addressing key challenges, such as long-term biosafety and scalable manufacturing. Future research should focus on developing more intelligent systems, such as multi-stimuli-responsive (e.g., pH/enzyme/redox) ‘logic-gated’ delivery platforms [106], which can further enhance therapeutic precision and safety. Among these, the ‘logic-gated’ drug release system represents a cutting-edge direction in current research. They are unlike traditional single-stimulus response systems, as such systems typically require multiple signals to combine under specific conditions to activate drug release, analogous to Boolean logic operations in electronic circuits [107]. Dosta et al. designed a nanoparticle system exhibiting dual sensitivity to matrix metalloproteinases and pH, wherein its linker rapidly degrades within the tumor microenvironment to trigger in situ nanogel formation. This prolongs drug retention time and enhances anti-tumor efficacy. This design concept demonstrates the potential for leveraging multiple characteristics of the tumor microenvironment to achieve precision drug delivery [108]. Its primary advantage lies in enhanced targeting specificity. By requiring multiple disease-specific signals to coexist, off-target drug release in healthy tissues is significantly reduced, ensuring therapeutic efficacy at pathological sites while minimizing systemic toxicity. Furthermore, such systems support temporally controlled combination therapies, enabling sequential release of different drugs through distinct logical operations within a single carrier [109]. This section explores two primary frontiers: Its transformative potential in biomedical therapy, exemplified by targeted cancer treatment, and its innovative use in intelligent packaging. Figure 8 illustrates the primary application fields of QCS, and for each domain, the discussion extends beyond the prospects to critically address the specific translational hurdles that bridge promising laboratory results and real-world implementation.

### 4.1. Anti-Tumor and Cancer Delivery Therapy

Additionally, studies have shown that quaternized chitosan derivatives exhibit outstanding antiviral activity against both enveloped and non-enveloped viruses, holding considerable promise as viable antiviral agents or disinfectants [110]. Studies have demonstrated the algicidal efficacy of quaternary ammonium compounds, further confirming their potential for preparing functional coatings. In such coatings, positively charged quaternary ammonium groups interact with negatively charged algal cells and inhibit their activity [111].

The distinctive acidic tumor microenvironment (TME) presents a key opportunity for targeted cancer therapy, for which the pH-responsive nature of QCS-based crosslinked networks is ideally suited. Leveraging dynamic covalent bonds (e.g., Schiff-bases) that cleave under acidic conditions (as discussed in Section 3.2.2), these networks can be designed for preferential drug release within tumors, enabling a paradigm shift from passive drug accumulation to active, site-specific delivery.

However, as described in Section 2.2.2 (Influence of Quaternary Ammonium Groups on Antibacterial Activity), quaternary ammonium groups that enhance cellular uptake also pose a risk of cytotoxicity at high degrees of substitution (DS) [40,42]. While increasing DS can improve efficiency, it may compromise biocompatibility. Therefore, to achieve a balance between therapeutic efficacy and biocompatibility, there is a need to seek strategies beyond simple DS modulation to minimize systemic toxicity while maximizing therapeutic effectiveness.

#### 4.1.1. Active Targeting to Decouple Uptake from Toxicity

A particularly effective strategy is to decouple ingestion efficiency from non-specific toxicity by employing active targeting. Studies have demonstrated that crosslinking chitosan-based materials with hyaluronic acid (HA) enables active targeting via tumor cell-overexpressed CD44 receptors, thereby significantly enhancing anti-tumor efficacy [104,105,106,107]. The mechanism of action of this specific recognition-targeting strategy between hyaluronic acid and CD44 is independent of whether chitosan is quaternized, making it applicable to delivery systems based on quaternized chitosan (QCS) as well [112].

In addition to ligand−receptor interactions, the inherent dynamic covalent bonds within the QCS network also contribute to improved targeting accuracy. In cancer therapy, the acidic tumor microenvironment (with a pH of approximately 6.5) can be exploited by designing QCS nanoparticles to remain stable at normal physiological pH (7.4) but undergo degradation or structural changes in the acidic tumor environment, thereby enabling targeted drug release. This site-specific action minimizes damage to healthy tissues and enhances therapeutic efficacy [113].

Dynamic covalent bonds within QCS networks, such as Schiff-bases (imine bonds) [114] and borate esters [115], are particularly advantageous for this purpose. These bonds are stable at neutral pH but undergo selective cleavage in the acidic environments of tumors (pH 6.5), inflammatory tissues, or cellular compartments like endosomes/lysosomes (pH 4.5–6.0). This cleavage triggers network dissolution or swelling, resulting in a rapid and accelerated release of the chemotherapeutic payload. For instance, curcumin-loaded chitosan-selenium nanocomposites (CUR-Cs-SeNCs) exhibited a release rate at pH 5.5 that was 1.83 times faster than at pH 7.4. Curcumin itself demonstrates greater stability under acidic conditions, synergizing with the properties of the tumor microenvironment to collectively elevate therapeutic efficacy [116].

Beyond dynamic covalent bond cleavage, emerging strategies for enhancing tumor penetration focus on intelligent surface charge modulation. In a comprehensive review by Zhang et al., charge-reversal nanoparticles leverage tumor microenvironment-specific triggers—such as acidic pH, redox gradients, and enzymatic activity—to dynamically switch surface charge from neutral/negative during circulation to positive within tumors [117]. This transformation enhances tissue infiltration and cellular internalization while minimizing off-target toxicity, offering a promising framework for engineering QCS-based nanocarriers capable of overcoming the transport limitations in solid tumors.

Structure−activity relationship studies further underscore the importance of degree of substitution (DS) optimization in these systems. Fan et al. [118] systematically investigated hydrogels of hyaluronic acid−chitosan conjugates (HTCC) with varying DS values and found that when DS = 41%, the gelation time, adhesion, and functional performance achieve an optimal balance. This finding reinforces the critical role of precise control over DS values in maximizing functionality while minimizing toxicity, a consideration that is equally applicable to anticancer formulations.

#### 4.1.2. Synergistic Combination Strategies

The integration of QCS with functional crosslinkers further expands the therapeutic toolbox while maintaining the DS toxicity balance. The curcumin-loaded quaternized alginate-g-poly(tetrahydrofuran) (QA-g-PTHF) amphiphilic graft copolymer exhibits a ninefold enhanced anticancer effect against HeLa cells compared to free curcumin [119]. Notably, this improved efficacy is accompanied by good safety: At pH 5.5 (simulating TME), the cumulative release rate approaches 90%, whereas the release rate is significantly reduced at pH 7.4 (simulating normal physiological conditions) [119]. This pH-sensitive release profile, coupled with curcumin’s inherent stability in acidic environments, is highly beneficial for cancer therapy [120]. By leveraging this pH-responsive mechanism, the QCS crosslinked network has facilitated a paradigm shift in anti-tumor therapy, transitioning from passive drug-loading to active targeting. In oncology applications, chitosan-based crosslinked networks significantly enhance anti-tumor efficacy by increasing local drug concentration at the tumor site and prolonging the duration of action [121].

Similarly, carboxymethyl konjac glucomannan/chitosan (CMKGM/CS) nanogels crosslinked via amide bonds achieved a 10% reduction in curcumin release and improved encapsulation efficiency within 8 h compared to uncrosslinked gels [78]. Such studies indicate that optimizing delivery dynamics can be achieved by adjusting the network structure, without relying solely on modifying the degree of substitution (DS), which is also a key principle for decoupling efficacy and toxicity. The therapeutic potential of these methods has been further validated through in vivo studies. In another study, CUR-Cs-SeNCs not only demonstrated enhanced release at pH 5.5 but also reduced tumor weight by 36.3% and 51.0% compared to the free curcumin group and saline control group, respectively [116]. The inherent stability of curcumin under acidic conditions [122] synergizes with the pH-responsive release mechanism of the QCS network to enhance therapeutic efficacy while minimizing systemic exposure to the greatest extent possible.

#### 4.1.3. Expanding the Therapeutic Repertoire

This is based on the previously discussed design principles, including active targeting, pH-responsive degradation, and synergistic co-delivery, which all address the limitations of traditional chemotherapy. Additionally, the study utilizes QCS as a hydrogel dressing to integrate these mechanisms, creating a moist wound environment that promotes cell migration and supports tissue regeneration, thereby providing a multi-dimensional solution for post-surgical cancer treatment or local management of skin malignancies [123]. Additionally, a significant application in drug delivery involves the synthesis of mucoadhesive nanoparticles created through the crosslinking of quaternized chitosan with alginic acid (FD), which are loaded with epigallocatechin gallate (EGCG). This system not only protects EGCG from degradation in neutral phosphate-buffered saline (pH 6.8) but also extends its release duration to over 300 min, thereby exhibiting excellent protective and sustained-release properties [124]. Concurrently, its cationic characteristics enhance cellular uptake, and as nanoparticles, QCS can effectively safeguard and deliver siRNA to tumor cells, making it suitable for gene therapy and nucleic acid delivery applications [125].

Beyond small-molecule drugs, QCS-based nanocarriers demonstrate considerable potential in therapeutic protein delivery. For instance, quaternized chitosan nanoparticles prepared via the tripolyphosphate ion gelation method and subsequently crosslinked with gardenoside successfully encapsulated bovine serum albumin (BSA) as a model protein drug [83]. This dual-crosslinking strategy not only achieved a high encapsulation efficiency of approximately 46% but also significantly prolonged the protein release profile. Compared to the uncrosslinked formulation, this fully demonstrates the suitability of QCS networks for sustained protein delivery. Furthermore, surface modification of chitosan nanoparticles with targeted ligands such as folic acid enhances the uptake of loaded chemotherapeutic agents by cancer cells, demonstrating the potential of targeted delivery strategies [126]. The integration of multiple functionalities into a single chitosan-based nanocarrier represents a powerful paradigm for theranostic applications. Wu et al. pioneered this concept by developing chitosan-based hybrid nanogels with in situ-immobilized CdSe quantum dots, achieving simultaneous optical pH-sensing, tumor cell imaging, and pH-regulated anticancer drug release [127]. The covalently crosslinked nanogels exhibited excellent structural stability, low cytotoxicity, and reversible pH-responsive behavior in the pathological pH range of 5–7.4, demonstrating the feasibility of combining biosensing, bioimaging, and therapy within a single chitosan-based nano-object. This foundational work has inspired subsequent developments in chitosan−carbon dot hybrid systems for NIR imaging-guided synergistic therapy. These examples illustrate the broad applicability of the QCS system in overcoming stability and delivery challenges for diverse therapeutic agents.

QCS has also demonstrated considerable potential as a vaccine adjuvant, capable of stimulating stronger humoral and cellular immune responses [128]. Nanoparticles encapsulating Newcastle disease virus (NDV) were prepared using chitosan (CS), hydroxypropyl trimethylammonium chloride chitosan (HACC/CS), and sulphated chitosan as adjuvants. The results indicated that NDV-loaded CS and HACC/CS nanoparticles induced qualified humoral immunity (HI > 5) and higher cellular immunity levels compared to traditional oil-in-water emulsion vaccines, achieving 100% protection against a highly pathogenic NDV challenge [129]. Chitosan quaternary ammonium salts also exhibit significant advantages in mucosal vaccine delivery via thermosensitive hydrogels. Wu et al. [130] were the first to report the use of HTCC/α,β-glycerophosphate thermosensitive hydrogels for H5N1 vaccine nasal mucosal immunization. This study confirmed that the system can significantly prolong antigen nasal retention time, enhance antigen transcellular transport, and induce potent systemic and mucosal immune responses.

The various application strategies discussed in this section collectively demonstrate that the fundamental trade-off between drug safety and toxicity identified in the section entitled Influence of Quaternary Ammonium Groups on Antibacterial Activity can be addressed through rational molecular engineering rather than simple compositional optimization. By integrating active targeting moieties, leveraging dynamic covalent chemistry, and combining with synergistic delivery agents, QCS-based systems can achieve the high drug safety required for efficient cellular uptake while minimizing off-target toxicity. However, these complex designs also present new challenges, including the need for precise control over conjugation chemistry, potential immunogenicity of targeting ligands, and difficulties in scalable production, which will be explored in Section 5.

### 4.2. Food Packaging and Personal Care Products

Beyond biomedical applications, QCS finds significant utility in food packaging and personal care, primarily driven by its potent and permanent antibacterial activity, excellent film-forming ability, and inherent biocompatibility. The permanently charged quaternary ammonium groups provide sustained antimicrobial protection, independent of environmental pH. Simultaneously, the polymer’s backbone allows for the formation of robust, functional matrices that can be integrated with natural indicators for smart packaging or act as effective carriers for cosmetic actives.

In addition, quaternary ammonium chitosan (QCS) also exhibits excellent inherent antibacterial properties due to its unique permanently charged structure. It not only retains the inherent biocompatibility and biodegradability of natural chitosan but also has broad application prospects in active packaging films and anti-aging skincare products.

#### 4.2.1. pH-Responsive Smart Packaging

pH-sensitive smart films represent an advanced class of materials used to monitor product freshness and quality in real-time. Chitosan and its derivatives are frequently employed as matrix materials for these intelligent systems due to their excellent biocompatibility, biodegradability, non-toxicity, and innate pH sensitivity [131].

A common strategy is to incorporate natural pigments. For instance, films containing anthocyanins exhibit clear color changes from red to green as pH increases, effectively monitoring pork and shrimp spoilage [132]. Other studies have utilized films made from methylcellulose (MC) and zinc alginate, incorporating anthocyanins (Anth) as freshness indicators, which exhibit enhanced thermal stability, mechanical properties, water resistance, and ultraviolet blocking capacity. These films achieved inhibition rates of 81.75% against *Staphylococcus aureus* and 84.19% against *Escherichia coli*. Most notably, when exposed to buffers of varying pH, the film underwent a distinct color transition from pinkish-red to blue, and finally to blue-green, effectively detecting spoilage in pork and shrimp [51].

Similarly, incorporating alizarin into chitosan films enhanced their antibacterial and antioxidant properties while enabling function as acid−base indicators. The films exhibited color changes from khaki to light brown, allowing effective monitoring of fish freshness and demonstrating potential for active and intelligent food packaging applications [133].

Curcumin serves a dual function within packaging films, acting as both an antimicrobial agent and a natural pH indicator. Research incorporating curcumin (Cur) into a tara gum/polyvinyl alcohol (PVA) mixed matrix demonstrated that as curcumin content increased from 0.1% to 0.5%, light transmittance at 600 nm decreased slightly, while oxygen barrier properties diminished and surface wettability increased. Most importantly, this smart film exhibited visible color changes within 1–3 min in an NH_3_ environment, with accelerated color shifts under elevated humidity, demonstrating its capability for real-time monitoring of shrimp spoilage [134].

The application of natural pH indicators in pH-sensitive responsive films faces numerous limitations, primarily manifested in their insufficient inherent stability [135], inability of the color rendering range to effectively cover the entire shelf life of food [136], and compatibility issues with film matrix materials [137]. Therefore, their performance can be further improved through other means.

The mechanical and functional properties of these smart films can be further enhanced through composite approaches. Incorporating turmeric oil (containing essential oils and pigments) and anthocyanin extracts into a chitosan matrix reinforced with α-chitin nanocrystals improved mechanical properties and hydrophobicity. These films exhibited near-total UV/Vis light blocking below 550 nm wavelengths while demonstrating antioxidant properties and distinct color changes upon exposure to ammonia gas and pH solutions, with more pronounced color shifts at higher turmeric oil concentrations [138].

Crosslinking strategies provide another avenue for optimizing packaging performance. Research incorporating curcumin into a chitosan (CS)/oxidized chitin nanocrystal (O-ChNCs) matrix demonstrated that adding O-ChNCs and Cur to CS films significantly enhanced both mechanical properties and barrier performance through strong interfacial interactions (electrostatic forces and hydrogen bonds). The resulting Cur-CN films exhibited pronounced color shifts (yellow to red) across a pH range of 3.0 to 10.0, enabling direct visual monitoring of fish freshness during application [139].

Advanced encapsulation techniques further improve functionality. Research encapsulating curcumin in succinylated soy protein isolate (SSPI) emulsion and combining it with chitosan (CS) to prepare smart films resulted in materials that blocked 97.11% of ultraviolet radiation, reduced water vapor transmission rate by 42%, and enhanced swelling degree (32.26%), water solubility (12%), thermal stability (70.79%), and elongation at break. The film exhibited significant antibacterial activity and released 82.60% of curcumin via Fickian diffusion, gradually turning red under alkaline conditions, demonstrating substantial potential for food freshness monitoring and packaging [140].

The integration of nanoparticles enhances pH-sensitive smart responsive films. Studies investigating the effects of nano-ZnO or CaCl_2_ on polyvinyl alcohol (PVA)/chitosan (CS)/purple tomato anthocyanin films revealed that both additives reduce film transmittance in the visible light spectrum. Films incorporating nano-ZnO exhibited heightened sensitivity to pH changes, while PCP-CaCl_2_ films demonstrated superior mechanical properties, antibacterial activity, antioxidant performance, and effectiveness as freshness indicators compared to PCP-ZnO films [141].

Multifunctional, multi-layer approaches represent the cutting edge of smart-packaging technology. A three-layer pH-sensitive active film based on furfural-derived polysaccharide (FUR), chitosan (CHIT), and hydrolyzed gelatin (HGEL) incorporated curcumin ethanol extract (CUR) enriched with lemongrass essential oil (LEO) into its middle layer. The addition of CUR-LEO altered surface coloration and enhanced UV-blocking properties, while the film’s antioxidant activity increased with rising CUR-LEO concentration. The colored film exhibited pronounced color shifts during carp storage, demonstrating efficacy in freshness monitoring [142]. These developments indicate that creating smart responsive film materials using chitosan and its derivatives represents a major focus for future research and development.

#### 4.2.2. Cosmetics and Personal Care Sector

Chitosan and its derivatives exhibit excellent adhesion, water resistance, cell compatibility, and UV absorption properties [143], presenting broad application prospects within the cosmetics sector [144]. Their inherent film-forming, moisturizing, and adhesive properties render them ideal components in cosmetic formulations, serving as natural conditioners and moisturizers for skin and hair [145].

In hair care applications, chitosan and its cationic derivatives interact with keratin to form transparent, elastic films on hair fibers. These films enhance hair strength and softness while preventing damage [146]. Accumulated research evidence demonstrates that incorporating chitosan, microcrystalline chitosan, and quaternized chitosan into shampoos and hair gels works well due to their excellent film-forming activity and moisturizing properties [145]. Additionally, chitin’s film-forming properties help prevent moisture loss in the skin, enhancing elasticity and smoothness, and making it suitable for moisturizing cosmetics [147].

Transparency is a particularly valuable characteristic for cosmetic applications, as it enables immediate observation of product quality and alterations. Han and colleagues developed a standard formula for quantifying this property:

(4) Transparency=A600X

where *X* denotes film thickness (mm) and *A* represents absorbance at 600 nm. Lower absorbance values correspond to higher transparency and lower opacity.

Chitosan-based delivery systems (nanoparticles, microcapsules, and hydrogels) significantly enhance the efficacy of cosmetic active ingredients by improving their stability, absorption, and targeted delivery [148]. Research has demonstrated that nanotechnology facilitates active ingredient absorption through skin layers. Utilizing chitin nanofiber−hyaluronic acid nanoparticles (CN-HA) with added active ingredients such as lutein enhances loading capacity and promotes penetration through skin layers, thereby improving efficacy and safety as anti-aging agents [149].

The regenerative properties of chitosan are valuable in anti-aging formulations. Research using chitosan gel combined with collagen and aloe vera demonstrated enhanced stability and the ability to induce local cell proliferation. The combination improved biocompatibility, adhesion, and proliferation of skin fibroblasts, ultimately increasing skin rejuvenation and regeneration capacity [150].

Chitosan-based delivery systems (nanoparticles, microcapsules, and hydrogels) significantly enhance the efficacy of cosmetic active ingredients by improving their stability, absorption, and targeted delivery [148,151,152]. Research has demonstrated that nanotechnology facilitates active ingredient absorption through skin layers. Utilizing chitin nanofiber−hyaluronic acid nanoparticles (CN-HA) with added active ingredients such as lutein enhances loading capacity and promotes penetration through skin layers, thereby improving efficacy and safety as anti-aging agents [149].

The regenerative properties of chitosan are valuable in anti-aging formulations. Research using chitosan gel combined with collagen and aloe vera demonstrated enhanced stability and the ability to induce local cell proliferation. The combination improved biocompatibility, adhesion, and proliferation of skin fibroblasts, ultimately increasing skin rejuvenation and regeneration capacity [150].

Chitosan’s UV-blocking capabilities make it a valuable component in sunscreens [153]. Research synthesizing HAp-chitosan gels by dissolving chitosan in phosphoric acid and adding hydroxyapatite (HAp) via coprecipitation resulted in materials that exhibited pronounced antibacterial effects and resistance to ultraviolet radiation, proving the potential of chitosan-HAp gel for skincare as an antibacterial sunscreen [154].

The antioxidant properties of chitosan can be enhanced through modification. Research employing an industrial-scale hydrothermal method synthesized chitosan−glucose derivatives (CG-MRP) via a simplified one-pot Maillard reaction process. Functionalizing chitosan with glucose at varying ratios produced derivatives that exhibited fivefold enhanced radical scavenging activity compared to pure chitosan, alongside improved sun protection efficacy (SPF 6.30 ± 0.01). These modified chitosans may serve as biodegradable photoprotective agents in skincare products and as preservatives within the food and cosmetics industries [155].

Beyond skincare, chitosan and its derivatives find applications in oral care for preventing and treating periodontitis and dental caries [156]. They also promote the regeneration of soft tissues, including skin, muscle, and nerves, and serve as effective wound dressings to promote healing [123]. Although the direct application of quaternary ammonium salt chitosan as a carrier for controlled-release drugs in anti-aging skincare products is still emerging, the existing research provides a strong theoretical foundation and technical support for this promising avenue.

## 5. Future Perspectives and Emerging Paradigms

Quaternary ammonium chitosan (QCS) crosslinked networks also hold considerable promise in biomedical applications. However, their clinical translation and large-scale production face major challenges, especially in terms of biosafety, process stability, and cost control [7].

### 5.1. Key Challenges in Translational Research

A primary challenge lies in the choice of crosslinker. Traditional chemical crosslinkers, such as glutaraldehyde, effectively enhance material mechanical properties but are severely limited by potential cytotoxicity and poor biocompatibility. Consequently, investigating naturally sourced, biocompatible crosslinking agents represents a critical research focus [157].

Natural crosslinkers such as genipin (extracted from gardenia fruit) exhibit significantly lower cytotoxicity than glutaraldehyde, possess excellent biocompatibility, and yield crosslinked networks with superior stability and mechanical properties. Research indicates that genipin-crosslinked chitosan-based materials can even exhibit anti-inflammatory properties, which is beneficial for applications like bone regeneration [158]. Furthermore, green crosslinking strategies utilizing cellulose derivatives (e.g., diacetylcellulose, DAC) and enzyme-catalyzed crosslinking (e.g., with laccase or transglutaminase) have garnered significant attention due to their favorable biodegradability and safety profiles [159,160]. These green crosslinking agents not only mitigate the toxicity risks associated with conventional alternatives but also provide novel pathways for developing next-generation smart delivery systems with enhanced biocompatibility and precise controllability.

A promising frontier is the design of “logic-gated” release systems. On this basis, enzyme-catalyzed crosslinking, as a mild, efficient, and environmentally friendly method, particularly has broad application prospects, effectively avoiding the toxicity and residue issues that may arise from chemical crosslinking agents. Employing enzymes like oxidoreductases or transglutaminases allows for precisely controlled crosslinking processes, yielding hydrogels with superior biocompatibility and tunable degradation behavior [161]. Notwithstanding these advantages, the optimization of enzyme-catalyzed crosslinking for complex QCS networks remains largely empirical. Addressing this challenge and achieving predictive material design necessitates a paradigm shift towards computational and data-driven approaches, such as molecular dynamics simulations and machine learning, to decipher the complex structure−activity relationships that govern network properties and drug release profiles.

In addition, advanced manufacturing technologies such as photopolymerization provide precise control over the crosslinking process in terms of time and space, making it possible to carry out crosslinking within specific time frames and areas. Notably, visible light crosslinking is particularly noteworthy due to its minimal damage to cells and tissues, demonstrating enhanced biocompatibility [162]. Future research should focus on developing novel visible-light photosensitizers to improve crosslinking efficiency and on integrating these techniques with 3D printing to fabricate scaffolds with complex, customized structures and functions. The recent development of 3D-printed stretchable cellulose hydrogels for wound healing underscores the practical viability of this approach for creating adaptive, patient-specific devices [163].

The integration of smart responsive materials is a key direction for future development and brings potential challenges. Combining QCS with photosensitive materials (e.g., gold nanoparticles, MXene) can facilitate the construction of photothermal-responsive drug delivery systems for combined chemo-photothermal therapy, effectively inhibiting tumor cell proliferation [164]. Alternatively, developing smart hydrogels responsive to multiple endogenous (e.g., pH, enzymes, and redox potential) and exogenous (e.g., temperature, light) stimuli will enable more precise and sophisticated drug delivery and treatment regimens.

For example, intelligent hydrogels can respond to multiple stimuli, such as temperature, pH, light, electric fields, and magnetic fields [165]. This “intelligent” characteristic gives hydrogels great potential in the field of drug delivery, enabling the precise release of drugs [166]. Additionally, by combining this with nanotechnology, such as gold nanoparticles, photothermal responsive systems can be designed to trigger thermal effects through external light exposure, thereby achieving drug release or synergistic therapy to effectively inhibit tumor cell proliferation. Furthermore, nanocarriers have been proven to effectively encapsulate bioactive compounds and efficiently deliver drugs to target cells by overcoming a series of intracellular barriers, achieving ideal therapeutic and diagnostic effects. At the same time, endogenous stimuli such as unique intracellular ATP, low pH, ion concentration, hypoxic environments, specific enzymes, or redox potentials can also act as signals to trigger drug release. These stimulus response strategies can be applied individually (single-stimulus response) or in combination (multiple stimulus response, cascade reaction) to achieve more precise and programmed drug release, thereby enhancing therapeutic effects and reducing side effects [167]. In summary, the development of smart responsive hydrogels and nano-delivery systems offers new hope in overcoming the challenges of traditional drug delivery, especially in fields like tumor treatment and management of infectious wounds.

### 5.2. Addressing Limitations of Green Crosslinking Systems

As highlighted in the Introduction, green crosslinking systems defined by verifiable metrics (zero organic solvent, high biodegradability, comparable mechanical/barrier properties, and natural origin) still face practical limitations that need targeted optimization for large-scale application. These limitations, including color change, prolonged reaction time, and cost concerns, have been partially addressed by existing research, providing feasible improvement pathways for future development.

#### 5.2.1. Color Limitation and Route to Improvement

Color change is a notable issue for some green crosslinked films, which may restrict their application in scenarios requiring high transparency or a specific appearance. Although OCMC-crosslinked films exhibit Δ*E*-related yellowing [102], this limitation can be mitigated by simple modification strategies. Blending with zein or reducing Schiff-base density has been shown to lower Δ*E* below 6% [53], which is within the acceptable range for most food packaging and biomedical applications where visual appearance is not a critical requirement. Future research could further explore the combination of different natural polymers or surface modification techniques to achieve both color optimization and retention of core functionalities such as mechanical strength and responsiveness.

#### 5.2.2. Reaction-Time and Kinetic Shortcut

Long crosslinking time is another factor restricting the industrialization of green crosslinking systems, as it increases production cycles and energy consumption. The 2 h thermal crosslinking process reported for OCMC-crosslinked systems [102] can be significantly shortened to ≤30 min by adopting pH-driven cold-setting technology [53] (Section 2.2). This shortcut not only improves production efficiency but also cuts energy input by more than 50%, aligning with the low-carbon philosophy of green manufacturing. Additionally, optimizing reaction parameters such as temperature, pH, and crosslinker concentration, or integrating microwave, ultrasound, and other auxiliary technologies, may further reduce reaction time while ensuring network structure stability.

#### 5.2.3. Cost Outlook at Lab-Scale vs. Industrial-Scale

Cost competitiveness is a key prerequisite for the commercialization of green materials. Current lab-scale OCMC dosage (4% *w*/*w* gelatin) translates to approximately $0.04 per m^2^ film (SI calculation; OCMC price $2.8 kg^−1^ [102], supplementary). This cost stays within the 5% premium tolerance reported for bio-packaging, indicating promising scalability. With the expansion of industrial production and optimization of raw material extraction processes (e.g., utilizing agricultural and forestry wastes for cellulose-derived crosslinkers), the cost is expected to further decrease. Moreover, developing multifunctional integrated green crosslinking systems that simultaneously achieve crosslinking, antibacterial activity, and responsiveness can reduce the need for additional functional additives, further lowering the overall production cost and enhancing market competitiveness.

Future research will continue to explore more efficient and safer response mechanisms combined with advanced manufacturing technologies. From a medical perspective, comprehensively evaluating the cytotoxicity, hemolytic activity, immunogenicity, metabolic pathways, biodistribution, and excretion profiles of these delivery systems in vivo is crucial for ensuring their clinical application safety. In-depth studies on drug release mechanisms, particularly the pH-responsive and temperature-responsive behaviors of quaternized chitosan networks and their controlled release effects on different drugs, will be key for designing the next generation of intelligent delivery platforms.

## 6. Conclusions

This review highlights the benefits of quaternized chitosan (QCS) networks in encapsulating and delivering active molecules. The intrinsic pH-responsive mechanism of these networks enables precisely controlled release across varying acidic and alkaline environments, providing crucial insights and tools for advancing intelligent packaging and pharmaceutical applications.

Quaternization fundamentally enhances chitosan’s properties by substantially improving its water solubility and positive charge density. This modification strengthens its binding affinity for negatively charged bacterial cell walls, thereby significantly boosting its inherent antimicrobial activity. As either a standalone antimicrobial agent or a functional drug carrier, QCS effectively enhances drug targeting and therapeutic efficacy. Through strategic crosslinking, QCS networks prolong retention time at specific sites (e.g., infection or tumor tissues), delivering sustained antibacterial and anti-inflammatory effects while responding to environmental pH shifts for intelligent drug release. This dramatically improves the stability and bioavailability of encapsulated active molecules, such as curcumin.

Furthermore, QCS-based nano-delivery systems exhibit enhanced cellular uptake efficiency and can be seamlessly integrated with other functional materials (e.g., hyaluronic acid for targeting, selenium for synergistic therapy) to construct multifunctional composite systems. These systems achieve simultaneous targeted delivery, real-time monitoring, and synergistic therapy. Critically, QCS materials maintain excellent biocompatibility and degradability even after modification, ensuring a high safety profile and presenting broad application potential in wound healing, tissue engineering, and biosensing.

Despite these advancements, the field is now confronted with the intricate challenge of decoupling the intertwined effects of crosslinking density, mesh size, and charge density to achieve truly predictive material design. Overcoming this challenge requires a paradigm shift from empirical optimization to rational design guided by computational modeling and machine learning. Furthermore, the potential of chitosan and its derivative systems is fully realized by advancing two key frontiers: The implementation of intelligent “logic-gated” release systems for unprecedented targeting specificity [70], and the resolution of practical limitations of green crosslinkers, such as reaction kinetics and coloration [102].

Notably, existing research has offered viable solutions to these limitations. For instance, blending with natural polymers can alleviate yellowing problems, pH-driven room-temperature curing can reduce reaction time, and industrial-scale optimization can lower production costs, thereby establishing a robust foundation for transformative applications.

Beyond these technical advancements, the translation of this promising technology still faces several persistent challenges that hinder its clinical and commercial implementation. Key among these are: (i) the precise control over crosslinked network parameters (e.g., mesh size, crosslink density); (ii) comprehensive long-term in vivo biosafety assessments; and (iii) the development of scalable, cost-effective manufacturing processes. Despite these hurdles, continuous innovation in green crosslinking techniques, coupled with deepening interdisciplinary collaboration between material science, pharmacology, and biomedical engineering, positions QCS-based intelligent delivery systems as having immense translational potential.

Future breakthroughs are anticipated in the integration of QCS systems with bioelectronic interfaces for closed-loop therapy, and in the development of fully biodegradable smart packaging that leaves no environmental footprint.

Thus, transitioning QCS-based systems from laboratory to clinical/commercial use depends on resolving long-term biosafety issues and establishing scalable manufacturing protocols.

## Figures and Tables

**Table 1:** Common chemical modification methods of chitosan and their impact on key properties.

Modification Method	Reaction Reagents	Reaction Site	Key Performance Changes
Direct/indirect quaternization [6,15]	GTA. CHPTAC.	C2-position amino group (-NH_2_)	Significantly enhanced solubility under neutral/alkaline conditions. Enhanced antimicrobial properties. Increased charge density. Expanded pH-responsiveness range.
Carboxymethylation [16]	Chloroacetic acid. NaOH.	C6 position, C3 position hydroxyl (-OH), C2 position amino group	Enhanced solubility under neutral/alkaline conditions. Reduced antibacterial activity. Enhanced hydrophilicity. Expanded pH response range. Enhanced metal chelation capacity.
Crosslinking [17]	Glutaraldehyde. TPP. Genipin.	The C2 amino group (-NH_2_) participates in the Schiff-base reaction.	Reduced solubility. Reduced antibacterial activity. Enhanced mechanical properties. Enhanced sustained release. Enhanced anti-swelling properties.
Graft copolymerization [18,19]	Acrylic PNIPAM. Azobenzene.	The C6 hydroxyl (-OH) or C2 amino (-NH_2_) group is activated to generate free radicals.	Hydrophilic monomers enhance solubility. Antimicrobial monomers enhance activity. Environmentally responsive.
Acylation [20]	Acetic anhydride. Benzoyl chloride.	The C2 amino group (-NH_2_, which reacts preferentially) and the C6 hydroxyl group (-OH, which participates under highly reactive conditions).	Reduced solubility. Reduced antibacterial activity. Controllable degradation rate.
Sulphate esterification [21]	Sulphuric acid reagent (e.g., SO_3_-pyridine).	C6 hydroxyl group (-OH, low steric hindrance, easily modifiable), C3 hydroxyl group (-OH, relatively low reactivity).	Enhanced solubility. Enhanced antimicrobial activity. Enhanced anticoagulant properties. Enhanced antiviral activity. Biocompatibility.
Blending/Compositing [22]	Cellulose. Microcrystalline cellulose. Nanocellulose. Polyvinyl alcohol (PVA) Starch. Montmorillonite essential oil. Plant extract.	The amino groups (-NH_2_) and hydroxyl groups (-OH) of chitosan form hydrogen bonds with the hydroxyl groups (-OH) of cellulose and PVA.	Enhanced solubility. Enhanced antimicrobial properties. Enhanced mechanical properties. Barrier property improvement. Controllable degradability.
Nanotechnology (Electrospinning/nanoparticles) [23]	Chitosan solution. PVA and other co-spun polymers. Acetic acid. WO3-x quantum dots. Au@SiO_2_. ZnONPs/GO.	Chitosan skeleton, which forms nanofibers through intermolecular interactions between polymer chains.	Reduced solubility. Enhanced mechanical properties. Enhanced drug-loading capacity and antimicrobial efficacy. Enhanced degradability.
Enzyme-catalyzed modification (e.g., laccase-catalyzed phenolic grafting) [24,25]	Laccase. Phenolic compounds (such as gallic acid, hydroquinone, resorcinol, 4-hexyloxyphenol). Hydrogen peroxide. Tyrosinase. Transglutaminase.	The amino group (-NH_2_) of chitosan and the phenolic hydroxyl group of phenolic compounds.	Reduced solubility. Enhanced antimicrobial activity. Enhance the mechanical properties and stability of composite materials. Enhanced antioxidant properties. Enzyme responsiveness. Enhanced degradability.
Biomolecular coupling (peptides, DNA) [26]	Antimicrobial peptides. Cysteine.	The amino group (-NH_2_) of chitosan; carboxyl group, amino group, thiol group, etc., of polypeptides.	Enhanced solubility. Reduced mechanical properties. Targeted recognition. Enhanced cell affinity. Degradability Enzyme-regulated degradation.

**Table 2:** Comparison of primary synthesis methods for quaternary ammonium chitosan (QCS).

Method Name	Reaction Mechanism	Common Reagents	Reaction Conditions	Advantages and Disadvantages
Direct quaternization [28,29]	The amino group (-NH_2_) of chitosan reacts directly with the epoxy/reactive group of the quaternization reagent to form a quaternary ammonium structure.	Glycidyl trimethylammonium chloride (GTA) 3-Chloro-2-hydroxypropyl trimethyl ammonium chloride (CHPTAC).	pH: 8–10. Temperature: 50–70 °C. Time: 4–6 h.	Highly efficient reaction with straightforward operation However, crosslinking agents may remain and require purification.
Epoxypropyl quaternization [30]	The epoxy group of epoxypropyl quaternary ammonium salts (e.g., EPTAC) undergoes ring-opening reaction with the amino group of chitosan, yielding quaternized products.	Epoxypropyl trimethyl ammonium chloride (EPTAC).	pH: 8–9. Temperature: 60–80 °C. Time: 6–8 h.	Highly substituted products exhibit remarkable water solubility, though elevated temperatures may induce side reactions.
Alkylation Quaternization [31]	Nucleophilic substitution reaction between amino groups and halogenated alkanes (e.g., methyl iodide) to introduce quaternary ammonium groups.	Methyl iodide (CH_3_I) Bromoethane (CH_3_Br).	pH: 10–12. Temperature: 30–40 °C. Time: 12–24 h.	Mild reaction conditions, Reagents readily available. However, low substitution degree and potential formation of by-products.
Reduction of Schiff-base [32]	Amino groups react with aldehydes (e.g., formaldehyde) to form Schiff-bases, which are then reduced to stable quaternary ammonium salts using reducing agents (e.g., NaBH_4_).	Formaldehyde Glutaraldehyde NaBH_4_.	pH: 4–6. Temperature: 50–60 °C. Time: 6–12 h.	Capable of introducing multifunctional groups. However, the procedure is complex and requires strict acid control.
Microwave-assisted quaternization method [33]	Microwave radiation accelerates molecular collisions between quaternization reagents and chitosan, reducing reaction time.	GTA. CHPTAC.	pH: 9–10. Temperature: 70–90 °C (microwave heating). Time: 0.5–2 h.	Rapid reaction with low energy consumption but requires specialized equipment; potential for localized overheating.
Enzyme-catalyzed quaternization	Utilizes enzymes (e.g., peroxidase) to catalyze the grafting reaction between chitosan and quaternization reagents.	GTA. CHPTAC + peroxidase.	pH: 6–8. Temperature: 30–50 °C. Time: 4–8 h.	Environmentally friendly and mild conditions. However, enzyme costs are high, and reaction efficiency is relatively low.

**Table 3:** Summary of encapsulation efficiency and release kinetic parameters of representative delivery systems based on chitosan and its derivatives.

Delivery System	Drug	Encapsulation Efficiency	Release Conditions	Release Rate Constant (*k*)	Release Exponent (*n*)	Mechanism
GTA-modified chitosan micelles [70]	Curcumin	~32%	pH 7.4	0.087 h^−n^	0.52	Anomalous transport (non-Fickian)
Quaternized aminated chitosan nanoparticles (Q-AmCs NPs) [77]	Curcumin	94.4 ± 0.91%	SGF (pH 1.2)/SCF (pH 7.4)	—	—	Slow release (54.0% cumulative at pH
Carboxymethyl konjac glucomannan/chitosan (CMKGM/CS) nanogels [78]	Curcumin	85–92%	PBS pH 7.4	0.095 h^−n^	0.61	Anomalous transport
N-Trimethyl chitosan (TMC) nanoparticles [79]	Ovalbumin (protein)	Up to 95%	PBS pH 7.4	—	—	Sustained release (>70% retained for ≥3 h)
Folic Acid-Conjugated Chitosan-Loaded Proanthocyanidin Nanoparticles (PC-CS/FA-NPs) [80]	Epigallocatechin gallate (EGCG)	72–85%	PBS pH 6.8	0.15 h^−n^	0.55	Anomalous transport
Trimethyl chitosan (TMC) nanoparticles [81]	α-Galactosidase (α-GAL)	~65%	pH 7.4 (physiological)/pH 5.0 (acidic)	—	—	pH-triggered release (stable at pH 7.4, releases at acidic pH)
Quaternized chitosan derivative nanoparticles (33% substitution) [82]	Insulin	52 ± 3%	Simulated intestinal fluid	—	—	Sustained release (>210 min)
Crosslinked chitosan/gelatin nanocomposite [67]	Dopamine	Not reported	pH 7.4 (neutral)	—	—	Higuchi kinetics (93% release in 24 h)
TPP/Genipin dual crosslinked QCS nanoparticles [83]	Bovine Serum Albumin (BSA)	46.37 ± 2.89%	pH 7.4 PBS	—	—	Sustained release (significantly prolonged after genipin crosslinking)

## Data Availability

No data was used for the research described in this review article. All information presented is based on previously published studies cited in the reference list. This paper is a narrative essay and does not involve data.
